# Characterizing the In Utero Phenome of the Chiari II Malformation—A Network Medicine Approach, Using Fetal MRI

**DOI:** 10.1002/pd.6741

**Published:** 2025-01-03

**Authors:** Hui Shi, Daniela Prayer, Joel Leinkauf, Johannes Tischer, Xu Li, Patric Kienast, Farjad Khalaveh, Julia Binder, Gregor Kasprian

**Affiliations:** ^1^ Department of Radiology Zhujiang Hospital Southern Medical University Guangzhou China; ^2^ Department of Biomedical Imaging and Image‐Guided Therapy Medical University of Vienna Vienna Austria; ^3^ Center for Medical Physics and Biomedical Engineering Medical University of Vienna Vienna Austria; ^4^ Department of Neurosurgery Medical University of Vienna Vienna Austria; ^5^ Department of Obstetrics and Feto‐Maternal Medicine Medical University of Vienna Vienna Austria

**Keywords:** biometric analysis, brain edema, Chiari II malformation, co‐occurrence analysis, phenotype

## Abstract

**Objective:**

To apply a network medicine‐based approach to analyze the phenome of the prenatal fetal MRI and biometric findings in the Chiari II malformation (CM II) to detect specific patterns and co‐occurrences.

**Method:**

A single‐center retrospective review of fetal MRI scans obtained in fetuses with CM II was performed. Co‐occurrence analysis was utilized to generate a phenotypic comorbidity matrix and visualized by Gephi software. Traditional univariate regression and geometric thin‐plate spline methodology were used to elucidate the mechanisms underlying the relationships between morphometric measurements and geometric landmarks of the spine, skull, and brain deformations.

**Results:**

The CM II phenome consists of 35 nodes interconnected by 979 edges with a density of 0.828. Key “hubs” identified within this network include spinal bony defects, reduced posterior fossa dimensions, and vermis ectopia. The brain edema phenotype appearing only in the fetal stage but disappearing after postnatal surgery, links to increased postnatal morbidity and demonstrates distinct shape patterns by geometric analysis. Traditional univariate regression reveals correlations among spinal defects, posterior fossa dimensions, and caudal extent of vermis ectopia. The degree of brain rearrangement versus spinal bony rearrangement shows a correlation (*r* = 0.721, *p* = 0.0023) by partial least‐squares analysis.

**Conclusion:**

The CM II prenatal phenome is a multifaceted network centered around three key elements—spinal bony defects, small posterior fossa, and vermis ectopia—with strong interconnections. Fetal brain edema emerged as an exclusively prenatally detectable and transient phenotype of prognostic relevance.

**Summary**

- What is already known about this topic?
  ◦ Spinal defects, small posterior fossa, and vermis ectopia characterize the Chiari II malformation.
- What does this study add?
  ◦ Network medicine combined with biometric analysis facilitates the exploration of in‐utero phenome development of the Chiari II malformation, utilizing fetal MRI data, validating prevailing theories on mechanical interactions between the spine, skull, and brain.
  ◦ The cerebral edema phenotype, while appearing transient, represents a more severe phenotype, exhibiting in this series a severer postnatal morbidity and disability.

## 1 Introduction

“Deep phenotyping” is increasingly demanded in prenatal diagnosis. Phenotyping helps to further exploit the ever‐expanding potential of prenatal genetic testing [1, 2]. In addition to prenatal ultrasound, fetal MRI has been shown to perfectly add to this concept by “deepening” the imaging characterization of specific malformations at prenatal stages of development.

Chiari II malformation (CM II) results from a complex interplay of developmental defects that emerge over time, affecting the formation of spinal and skull bones, the subarachnoid space, brain blood vessels, and surrounding structures, beginning as early as the embryonic stage. These interconnected anomalies lead to a range of brain development abnormalities [3], emphasizing the multifactorial nature of this disorder.

Among fetuses diagnosed with CM II, remarkable phenotypic variability has been documented, both on neurosonography as well as MRI [4, 5, 6], including normal to severely enlarged cerebral lateral ventricles [7, 8, 9, 10], a normal, thin, and/or abnormally shaped to a partial agenetis of the corpus callosum [11], little to severe ectopia of the cerebellar vermis, and various levels and dimensions of spinal defects. Furthermore, in previous studies, fetal brain edema/hemorrhage was characterized as a different phenotypes [12, 13, 14, 15] with prognostic relevance with white matter volume loss/hypoplasia [16]. The extreme grade of heterogeneity of phenotypic features in CM II led to the difficulty in associating diverse fetal imaging findings with certain short‐ or long‐term outcomes. Consequently, prenatal counseling of individual cases of CM II is still marred by hidden biases and excess variability [17]. Considering the inherent variability in motor and cognitive neurodevelopment, with symptoms ranging from subtle to life‐threatening [18], it becomes imperative to identify complex but specific phenotypic associations and morphologic interactions which might impact decision‐making and optimize case selection for prenatal therapy [7]. The vast variability and complexity in disease‐related phenotypes and their hidden interdependencies—however—constitute a major challenge in optimizing prenatal care. Standard clinical epidemiological approaches involving univariate and multivariate regression are not helpful in that regard, as they are confounded by a priori assumptions in grouping multiple subtypes.

Network medicine (NA) offers invaluable tools for navigating the complex relationships between disease components, promoting a shift away from viewing them as isolated elements [19]. By embracing a network‐centric approach, we can effectively discern and explore hidden connections between disease components, contributing to the construction of a more comprehensive and nuanced model of complex diseases. Correlation‐based networks have found utility in diverse contexts, including gene expression data analysis [20, 21], disease‐related phenotypes [22], and in the analysis of multiple quantitative phenotypes within complex diseases [23]. Nevertheless, the application of network medicine in fetal MRI phenotyping remains unexplored. By capturing the morphological complexity of various phenotypes, the imaging‐based phenome could offer new insights into the developmental mechanisms underlying CM II during the fetal stage.

This retrospective MR imaging data analysis was designed to use NA and morphometric tools in a large historical cohort of prenatally diagnosed CM II patients—partly with long term follow‐up data—to identify the core features of this common brain malformation and its interdependencies and relations to outcome data in a temporal manner. By identifying distinct phenotypic patterns and associations, we aimed to characterize the “phenome” of CM II pre‐ and postnatally and elaborate possible implications for postnatal outcomes in this cohort. Finally, we tried to refine this analysis and improve our understanding of the mechanical evolution underlying the observed comorbidity between the spine, skull, and brain rearrangements by applying biometric analysis.

## 2 Materials and Methods

### 2.1 NA Analysis and Data Visualization

NA was used to construct the “phenome” of CM II through a co‐occurrence algorithm utilizing prenatal MRI data. The network is undirected and weighted, with the nodes representing phenotypes that have appeared in the study cohort of CM II fetuses. The weight of each link corresponds to the number of fetuses in which the two phenotypes co‐occur.

### 2.2 Source Data and Study Populations

This retrospective single‐center study was approved by the institutional internal review board (Ethics Committee number 1716/2017). Inclusion criteria for the study were the presence of CM II—as characterized by the lemon configuration of the fetal head, small posterior fossa, vermis ectopia, and reduced external CSF spaces [24, 25, 26, 27]. Due to the local center's long‐standing history of fetal imaging, we retrospectively evaluated our database of over 8000 fetal MRI examinations conducted between January 1, 2007, and December 31, 2022. We included cases that met the criteria for CM II and were confirmed as open spinal defects through postnatal or postmortem imaging or autopsy. In vivo fetal MRI was performed using 1.5‐T or a 3.0‐T scanners (Philips Ingenia or Achieva with a 32‐channel body coil; Philips Medical Systems). Only cases with adequate quality and standard alignment for each structure measurement were included. To showcase the natural dynamic evolution of the in‐utero phenome of CM II without the influence of fetal surgery, we excluded fetuses who underwent prenatal surgery. In addition, a cohort of 50 postnatal CM II cases who underwent MR exams (28/50 were the same as those with prenatal MRI, 22/50 cases without fetal MRI) were included in the postnatal phenome analysis.

### 2.3 Phenotypic Selection and Co‐Occurrence Matrix Construction

A systematic analysis of CM II and spinal defect characteristics was conducted [4, 26] (including detailed qualitative and quantitative assessment of morphological deformation findings in the brain supratentorial and infratentorial structures, spine, skull, brain vasculature, CSF system, and other observed/present anomalies) by two experienced fetal neuroradiologists (D.P. and H.S., with 35 and 6 years of experience in fetal imaging, respectively) as summarized in Table S1 and recorded in a CSV file for each fetus. Interrater variability analysis was performed.

**FIGURE 1:**
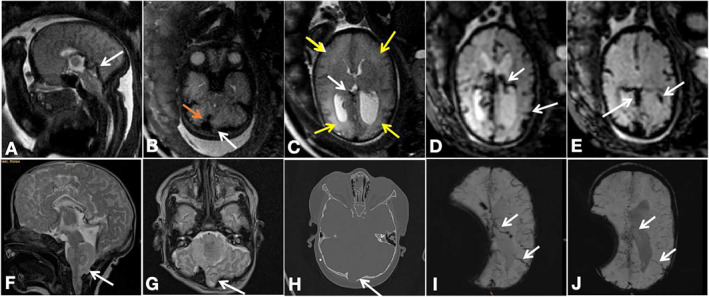
Phenotype of Chiari II malformation with brain edema, venous congestion, and intracranial hemorrhage. (A–E) Prenatal MRI performed at 35 + 3 weeks. (A) T2‐weighted images show a dilated straight sinus. (B) The herniating cerebellum protruding posteriorly to the occiput bone, the transverse sinus is compressed and narrowed (white arrow), and the adjacent dilated torcular (orange arrow) was noted. (C) Brain parenchyma edema (yellow arrows) and congestion in the deep ventricular veins (white arrow) were identified. (D, E) EPI‐T2* images show congested internal cerebral vein, superficial cortical veins, and ependymal hemorrhage at the left posterior horn (white arrows). (F–J) Postnatal MRI (after postnatal MMC repair surgery and EVD shunt) also demonstrates the protruding cerebellum (F, G) and the adjacent skull thinning like a notch shown on CT (H). (I, J) Follow‐up postnatal MRI performed at 3 weeks of age shows the congested cortical veins and deep veins at first (E, white arrow) and then, released at a subsequent scan performed at 23 months of age (F).

**FIGURE 2:**
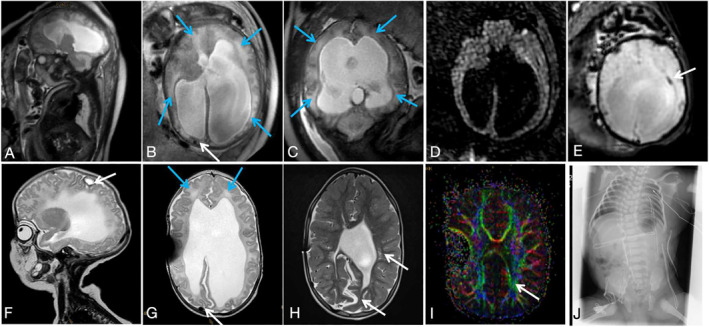
Phenotype of Chiari II malformation with brain edema and intracranial hemorrhage. (A–E) Prenatal MRI performed at 37 + 2 weeks shows the herniating cerebellum protruding posteriorly to the occiput bone (A), global brain parenchyma edema (blue arrows in B and C), and shifting and compressed superior sagittal sinus (B, white arrow). No hyperintensity region is shown on the DWI sequence within the parenchyma (D), indicating vasogenic edema, and the EPI‐T2* image shows ependymal hemorrhage at the left posterior horn (arrows in E). (F, G) Follow‐up postnatal MRI performed at 7 weeks of age (after postnatal MMC repair surgery and EVD shunt) shows cystic lesions with blood breakdown products (F, arrow), residual edema (blue arrows), and the shifting superior sinus (G, arrow). (H) Postnatal MRI performed at 5 years of age, shows a typical absence of the septum pellucidum and hemispheric interdigitations, periventricular white matter dysplasia, and volume loss (arrow). (I) DTI showed dysplasia of the white matter (arrow). (J) An X‐ray performed at 2 months of age showed hemivertebrae formation, bifurcated ribs, and aplasia of the 12th pair of ribs, as well as scoliosis.

Brain supratentorial edema was retrospectively identified by G.K. and H.S. (with 20 and 6 years of experience in fetal imaging, respectively) based on the following characteristics: visually higher signal intensity on T2‐weighted fast spin echo (T2W‐FSE) sequences as compared to the age‐matched normal brain performed on the same scanner, blurring of lamination on T2‐weighted or plus T2‐Flair sequences and effacement/depleted of external CSF spaces [14, 28] (Figures 1A–E and 2A–E).

Anatomical structure comparison among CM II fetuses with and without brain edema, including outer CSF spaces (degree of shrinking), maximum atrium width, third ventricle width, the cross‐sectional area of the superior sagittal sinus, maximum diameter of the posterior fossa, defect size, and caudal extent of vermis ectopia, were assessed using the T2‐TSE sequence (Figure S1).

The CSV file containing all the phenotypic information for the study cohort was put into Matlab R2021b for co‐occurrence analysis [29]. This analysis specifically focused on identifying patterns of phenotypic comorbidities (pairwise frequency) within each CM II case. We replicated the process to construct a postnatal phenotypic network using more comprehensive postnatal follow‐up data (MR/CT/X‐ray/ultrasound/clinical documents, Figures 1F–J and 2F–J) separately to examine the evolution of the network from the prenatal to the postnatal period. The follow‐up period ranged from 7 days to 21 years.

### 2.4 Visualization of Phenotypic Feature Co‐Occurrence Network and Analysis

**FIGURE 3:**
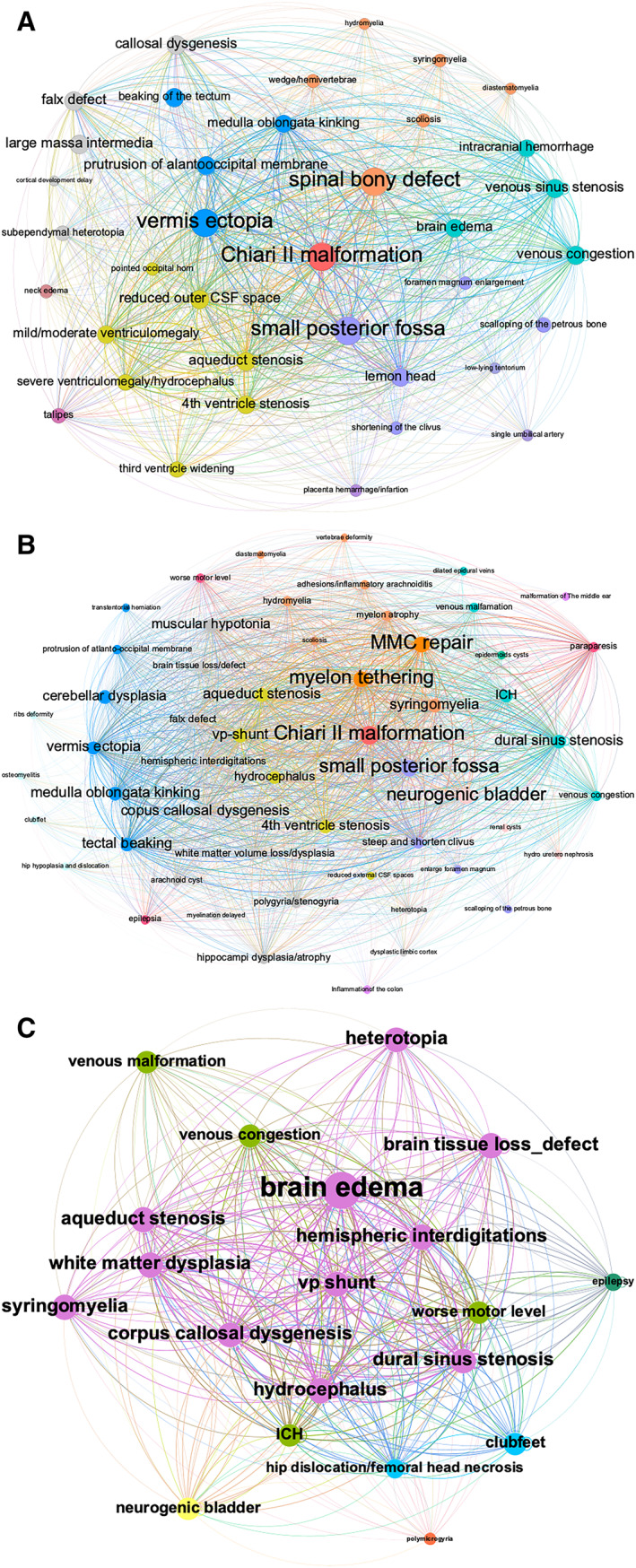
Visualization of the co‐occurrence network of Chiari II malformation phenotypes by Gephi. (A) The CM II phenome network in the fetal stage consists of 35 distinct diagnostic types and 979 co‐morbidity relationships, creating a very dense network that has been segmented into five communities, delineating the skull, brain (infratentorial/supra tentorial parenchyma, venous structure), spine, and CSF system. Within this network, spinal bony defects, small posterior fossa dimensions, and vermis ectopia prominently emerge as “hubs.” (B) Based on more comprehensive postnatal follow‐up data, the CM II phenome network in the postnatal stage demonstrates more phenotypes, including 47 unique phenotypic nodes and 1079 co‐morbidity relationships, and network density is 0.623. Unlike the prenatal network, MMC repair and spinal cord tethering emerge as “hubs,” showing the largest node size. These hubs are associated with phenotypes involving the brain, spine, intestine, genitourinary system, and musculoskeletal system, each marked in different colors. (C) Using the outcome data of a subgroup of 17 cases with prenatally identified brain edema, which emerges as a noteworthy phenotype with prognostic associations with ICH, epilepsy, worse motor level, and white matter volume loss/dysplasia.

Subsequently, this co‐occurrence matrix (CSV files containing node and edge data) was visualized as a co‐occurrence network graph using Gephi software (https://gephi.org) [30]. In this network, “Degree” was used to determine node size [19, 23], where larger nodes indicated more connections and represented a higher degree of co‐occurrence with other features. The weight of each edge corresponded visually to the thickness of the connecting lines and represented the pairwise frequency of co‐occurrence (Figure 3A). In addition, diseases were required to have a minimum co‐occurrence in two patients to avoid noise from rare events, such as the omission of micrognathia and diaphragm herniation.

The structural characteristics of the phenotypic co‐occurrence network were analyzed from both global and local perspectives. At the global level, network features, such as the number of nodes and connections, network density (defined by the portion of all possible connections in a network that are actual connections), and shortest paths (determined as the path traversing the lowest number of edges, hence making the fewest hops between nodes) [31, 32], were analyzed. At the nodal level, degree centrality, closeness centrality, and betweenness centrality were computed to uncover the relationship between phenotype features. Depending on the specific measure used, centrality means a network is directly connected to many others (degree centrality), close to many others indirectly (closeness centrality), or serves as a key broker between many other nodes (betweenness centrality) [33].

### 2.5 Traditional Biometric (Morphometric Measurements) Analysis of the Spine, Skull, and Brain

Relationships among morphometric measurements including inner and outer CSF spaces, diameter of the posterior fossa, defect size, and caudal extent of vermis ectopia were examined using repeated univariate regression. Morphologic differences were compared between CM II fetuses with and without brain edema. Analyses were considered significant at *p* < 0.05. UMAP Dimensional Reduction Analysis was conducted to discover cluster patterns within the dataset classified by CM II with or without edema. Statistical analysis was conducted using R version 4.0.5 (R Core Team).

**FIGURE 4:**
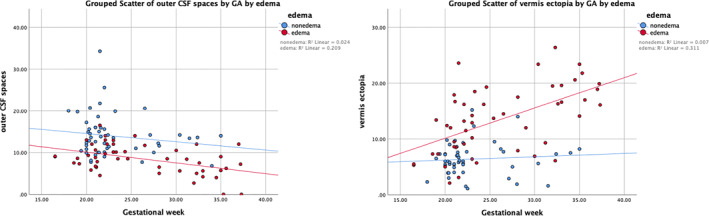
Grouped scatterplot of outer CSF spaces and caudal extent of vermis ectopia by gestational weeks. A linear decline in outer CSF space width throughout the investigated gestational weeks is shown among fetuses in the edema group, with an *R* ^2^ value of 0.261, while a linear increase in the caudal extent of vermis ectopia is shown with increased gestational weeks, with an *R* ^2^ value of 0.311.

A grouped scatterplot was created to display anatomical measurements based on gestational weeks (Figure 4).

### 2.6 Geometric Morphometric Analysis of Shape

NA can demonstrate, but not fully explain, the interconnections between phenotypes—primarily spine, skull, and brain deformations. To validate the hypothesis of these interconnections and their role in the mechanical evolution of in‐utero shape/phenotype, we employed thin‐plate splines and partial least‐squares analysis. These techniques help elucidate anatomical differences by tracking changes in landmark positions [6, 34, 35].

**FIGURE 5:**
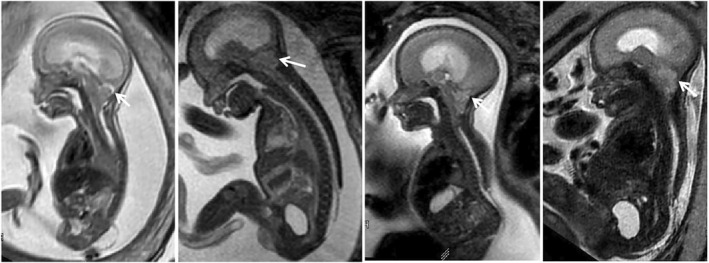
Examples of selected images from CM II cases specifically highlight all landmarks within a single sagittal slice. Shape variations are noted, encompassing the spinal defect location (ranging from lower thoracic to sacral) and size, posterior fossa area, inner and outer CSF spaces, and extent of vermis ectopia. Note the abnormally low tentorium inserts, placing the torcular herophili (white arrows) just above the rim of the foramen magnum—easy to be compressed.

A subset of mid‐sagittal images from CM II cases was selected, specifically highlighting all landmarks within a single sagittal slice (Figure 5). A TPS file of images was built with tpsUtil (http://www.sbmorphometrics.org) and imported to TPSDig.

**FIGURE 6:**
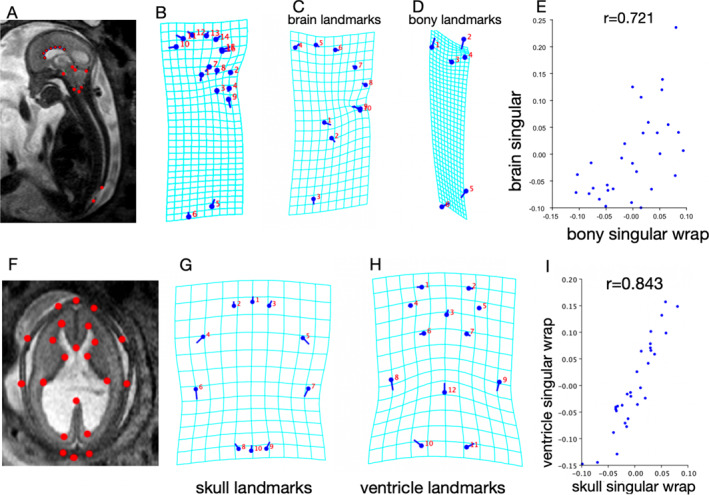
Geometric morphologic shape analysis of Chiari II malformation in fetuses. (A, B) Six bony landmarks (the top of the dorsum sellae 1, the basion 2, the opisthion 3, the torcular 4, the upper 5 and caudal limit 6 of the spinal defect)—and three hindbrain landmarks—the most rostral portion of the brain stem 7, the top of the tentorium 8, and the most caudal extent of the vermis 9, as well as a curve delineating the corpus callosum generated by seven continual landmarks 10–16, are marked on mid‐sagittal T2‐TSE images using TPSDig. (B) Shape variability among CM II cases is visualized by wireframe transformation grids and lollipop diagrams, where dots show the mean shape and lines show shape variation. (C, D) Wireframe transformation grids of brain landmarks (the most rostral portion of the brain stem 1, the top of the tentorium 2, and the most caudal extent of the vermis 3, as well as a curve delineating the corpus callosum 4–10 in D) and bony landmarks (the top of the dorsum sellae 1, the basion 2, the opisthion 3, the torcular 4, the upper and caudal limits of the spinal defect 5–6 in E), separately, show the deformation pattern on the analyzed bony and brain landmarks. Note the deformity of the grid centered on the point of the torcular. (E) Scatterplot of the degree of brain rearrangement versus bony rearrangement; the correlation is *r* = 0.721, significant at the 5% level. Each dot represents a single CM II fetus. (F) Ten skull landmarks and 12 ventricle landmarks delineating the contour of the skull and side ventricle are marked on axial T2‐TSE images at the falx level. (G, H) Wireframe transformation grids show mean shape and shape variability of the skull (G) and ventricle (H). (I) The Scatterplot depicting the degree of skull rearrangement versus ventricle rearrangement reveals a strong correlation, as indicated by *r* = 0.843.

TPSDig was used to mark six “bony” landmarks and three hindbrain landmarks, as well as a curve delineating the corpus callosum generated by seven sequential landmarks (Figure 6A). These landmark coordinate data for all age‐matched images were overwritten into the existing Tips file, and imported to MorphoJ. This step was duplicated on the landmarking to delineate the skull and ventricle contour [8] (Figure 6F).

Subsequently, seven steps were taken to perform shape analysis using MorphoJ: (1) a Procrustes fit was conducted with the Tips file including morphometric data sets; (2) data outliers were created by linking the biological landmarks; (3) a covariance matrix was generated; (4) classifiers (edema or nonedema) were added to the label of morphometric data; (5) PCA was used to show the major shape variations that maximized the separations between groups and how distinct the groups were; (6) the mean shape and shape variations in wireframe transformation grids and lollipop diagrams were visualized; and (7) partial least‐squares analysis was performed.

## 3 Results

### 3.1 Network Descriptions and Analysis

A total of 91 fetuses who underwent 101 MRI scans at a median gestational age of 24.4 weeks (interquartile range, 10.1 weeks) were included (Table S2). However, 21 cases were excluded due to loss of follow‐up (7/21), undergoing in‐utero surgery (5/21), the presence of additional congenital malformations (3/21), or inadequate image quality for performing morphometric measurements (6/21).

The prenatal phenotypic network—the “phenome” was constructed and visualized based on these real patient data (Figure 3A), identifying 35 distinct diagnostic features and 979 co‐morbidity relationships, resulting in a very dense network (density = 0.828). The phenomes were highly connected, with the average shortest path of 1.2. The majority of shortest paths were connected through spinal bony defects, small posterior fossa, and vermis ectopia based on shortest path analysis, suggesting that these three distinct features emerged as highly connected “hubs” that held the whole network together [36, 37, 38]. Their closeness centrality, degree centrality, and betweenness centrality occupied the top three (Table S3), indicating their strong co‐occurrence and a great influence on the phenome. To enhance the visualization of phenome modularity, the network was manually segmented into five communities encompassing the skull, brain (infratentorial/supratentorial parenchyma, venous structure), spine, and CSF system. Representative prenatal/postnatal phenotypic variables are illustrated in Figures 1 and 2 and Figures S2–S12.

Compared to the prenatal CM II network, more phenotypes emerged in the postnatal stage (underwent postnatal MMC repair surgery), resulting in 47 unique phenotypic nodes and 1079 edges of relationships, with a network density of 0.623 (Figure 3B). Unlike the prenatal network, MMC repair and spinal cord tethering emerged as central hubs, with more complicated phenotypes emerging that involved the brain, spine, intestine, genitourinary system, and musculoskeletal system.

Significantly, the brain edema phenotype was exclusive to the prenatal phenome. Subsequently, we correlated the prognostic outcomes of this newly defined phenotype with postnatal follow‐up data in this subgroup of 17 cases (Figure 3C). These outcomes encompassed intracranial hemorrhage (ICH) and brain tissue loss/defect (Figure S11), epilepsy, worse motor levels [39], and white matter volume loss/dysplasia [14, 15].

### 3.2 Traditional Biometric Analysis of the Spine, Skull, and Brain Further Elucidates the Interaction of “Hubs” in the Phenome

Interrater agreement regarding the morphologic measurements was *k* = 0.89–0.95, *p* < 0.001.

There were intercorrelations observed among the values of outer CSF spaces, maximum atrium width, third ventricle width, maximum diameter of the posterior fossa, cross‐sectional area of the superior sagittal sinus, defect size, and caudal extent of vermis ectopia (Tables 1, 2, 3, 4).

**TABLE 1:** Comparison of means: Chiari II with brain edema versus without edema.

Measure	Mean, CM II with edema	Mean, CM II without edema	Two‐tailed *p*‐value by *t*‐test
Gestational week	26.1	22.6	0.003
Outer CSF spaces (mm)	8.5	14	< 0.001
Atrium (mm)	18.1	13	< 0.001
Third ventricle width (mm)	6.3	3.6	< 0.001
Spinal defect size (mm)	30.5	15.9	< 0.001
Posterior fossa width (mm)	21.4	26.0	< 0.001
Caudal extent of vermis ectopia (mm)	13.4	6.4	< 0.001
Cross‐sectional area of superior sagittal sinus (mm^2^)	5.1	4	0.076

**TABLE 2:** Correlation between spinal defect size and other measurements.

Correlation between defect size and …	Pearson' *R*	Two‐tailed *p*‐value
Outer CSF spaces	−0.320	0.001
Atrium	0.553	< 0.001
Third ventricle width	0.416	< 0.001
Posterior fossa width	−0.544	< 0.001
Caudal extent of vermis ectopia	0.553	< 0.001
Cross‐sectional area of superior sagittal sinus	−0.410	< 0.001

**TABLE 3:** Correlation between posterior fossa width and other measurements.

Correlation between posterior fossa width and …	Pearson' *R*	Two‐tailed *p*‐value
Outer CSF spaces	−0.313	0.001
Atrium	0.479	< 0.001
Third ventricle width	0.732	< 0.001
Caudal extent of vermis ectopia	0.527	< 0.001
Cross‐sectional area of superior sagittal sinus	0.603	< 0.001

**TABLE 4:** Correlation between vermis ectopia and other measurements.

Correlation between vermis ectopia width and …	Pearson' *R*	Two‐tailed *p*‐value
Outer CSF spaces	−0.511	< 0.001
Atrium	0.582	< 0.001
Third ventricle width	0.542	< 0.001
Cross‐sectional area of superior sagittal sinus	−0.286	0.004

A 2D UMAP dimensional reduction analysis illustrated distinct cluster patterns between the CM II with and without brain edema phenotypes (Figure S13).

The width of outer CSF spaces decreased linearly in fetuses with edema across gestational weeks (*R* ^2^ = 0.261), while the caudal extent of vermis ectopia increased linearly with gestational weeks (*R* ^2^ = 0.311) (Figure 4). No other measurements showed a clear trend.

### 3.3 Geometric Morphometrics Analysis of Shape Validates the “Deformation” Sequence Diathesis of CM II Phenome

Fifteen mid‐sagittal T2‐TSE images of fetuses with CM II and brain edema were selected, capturing not only the mid‐sagittal brain but also associated spinal defects (Figure 5). Additionally, 15 age‐matched CM II cases without edema were included. All selected images clearly delineated the relevant anatomical landmarks (Figure 6A). Procrustes shape coordinates represent the relative positions of points and are visualized by a lollipop diagram and wireframe transformation grids (Figure 6B).

The principal component (relative wrap) plot shows the approximate position of the centroid of the CM II cases with and without brain edema, and the distinct shape pattern between groups is notable (Figure S14). A partial least‐squares analysis scatterplot of the degree of brain rearrangement versus spinal bony rearrangement (Figure 6C–E) shows the correlation (*r* = 0.721, *p* = 0.0023) between the extent to which the brain is wrapped and the extent to which the bone is wrapped (specifically, concerning displacement of the opisthion of the posterior fossa). The correlation between shape changes in the skull and ventricle (Figure 6F–I) in CM II fetuses is indicated by *r* = 0.843 (*p* < 0.0001).

## 4 Discussion

In this study, we addressed the heterogeneity and complexity encountered in CM II by constructing a phenotypic co‐occurrence network based on fetal MRI diagnostic features. This novel approach facilitates the identification of phenotypically essential associations and their potential impact on postnatal outcomes. Spinal defects, skull deformation, and vermis ectopia emerged as core connection “hubs” in the prenatal CM II phenome, with degree centrality, closeness centrality, and betweenness centrality occupying the top three, and playing a significant role in contributing to the overall heterogeneity. The cerebral edema pattern emerged as one of the most important associations of the CM II phenome in the cohort that did not undergo fetal surgery, appearing transiently only in the fetal stage and was associated with more postnatal comorbidities. To better characterize this phenotype, we employed geometric shape analysis to explore the potential mechanical forces acting on landmarks in the spine, brain, and skull. This analysis supported the hypothesis of a “deformation sequence” diathesis in CM II, as evidenced by interconnections revealed within the phenome network. Consequently, this phenotype may serve as a potential MR biomarker indicative of the severity of supratentorial involvement.

The algorithmic and self‐organizing process of human development cannot be understood by exclusive genomic analysis alone. Phenome‐level analysis is nowadays a key component of modern evolutionary developmental research [40] and helps to characterize the continuous unfolding of transient developmental morphologies in a dynamic fashion, respecting the complexity of the observed developmental process. However, this tool has not been extensively exploited in clinical prenatal medicine. One strategy is to perform data mining of phenotypically rich data sources — the complex deformation of CM II presented in the local center's long‐standing history of fetal imaging. The extremely high density of the phenome network suggests that phenotypes generally have strong co‐morbidities underlying mechanical interaction with each other. According to McLone and Knepper's unified theory of CM II [41], the open neural tube defect allows CSF to drain through the central canal and is therefore not maintained in the ventricular system, which results in a “lemon shaped head” with a pointed shape of the occipital horn [9]. Mechanical effects induce failure to distend the third and lateral ventricles (Figure 6F–I), which is also needed for neuron migration [3] and calvarial development. Without this ventricular CSF driving force, the posterior fossa never fully develops. With later rapid growth of the rhombencephalon, the cerebellum primarily develops in an atypical position—most elegantly described as “vermian ectopia,” rather than “herniation” (although active mechanical displacement may contribute during advanced gestation). Polymicrogyria, enlargement of the massa intermedia, a low‐lying tentorium, and base‐of‐skull anomalies are produced by the same lack of ventricular distention in the telencephalon. The intrinsic lack of movement caused by spina bifida may lead to skeleton deformation—clubfeet and scoliosis—associations as demonstrated by the phenome. Like the open neural tube defect, the anatomical disturbance varies in degree from child to child [7]. In this sense, spinal defects, skull deformation, and vermis ectopia are leading deformations and their interactions may give rise to a range of complex phenotypes. This theory is particularly apt for explaining the role of the “core hubs” in the phenome network. The ensuing deformations—including those of the skull, brain, venous structures, CSF dynamics, and skeletal system—are linked to a primary defect: spinal bifida malformation, a pathogenetic sequence that has occurred.

Moreover, upon comparing prenatal and postnatal phenomes, it becomes evident that brain edema manifests as a transient phenotype exclusively during the fetal stage, gradually diminishing postnatally (Figures 1 and 2), and likely attributable to postnatal repair surgery, which could be a sign before irreversible brain parenchymal damage similar to the “presyrinx lesion” in the spinal cord [42]. Given the novelty of the brain edema phenotype, we have delved into its potential prognostic relevance with postnatal clinical morbidity and disability, and its origination from vascular changes, specifically dura sinus stenosis and venous congestion. This correlation is further supported by follow‐up outcomes of this cohort, encompassing postnatal intracranial hemorrhage, epilepsy, worsened motor function, and white matter dysplasia within the cohort, visualized in a network. Using the brain edema phenotype as an example, we aim to shed further light on the mechanical factors influencing fetal development. By applying shape analysis as an independent, complementary method, we seek to enhance our understanding of this association.

First, traditional biometric measurements indicated a larger defect size, smaller posterior fossa dimension, and a deeper extent of vermis ectopia in the brain edema phenotype (Table 1). In addition, 2D UMAP analysis based on anatomical measurements further illustrated distinct clustering patterns between the with and without brain edema phenotypes, hinting that morphometric variations collectively contribute to the formation of the brain edema phenotype. This observation was reinforced by correlation analysis in which defect size seemed to be an important factor that could explain the variability of the posterior fossa width, the extent of vermis ectopia, and inner and outer CSF spaces. Vermis ectopia as well as outer CSF spaces influenced the dura sinus size (Tables 2, 3, 4), which suggests the possibility of a common etiology [10, 43]. Second, to elucidate multi‐dimensional structural variability and inter‐correlation, we employed shape analysis. The results indicate that CM II is a complex condition where diverse morphological abnormalities synergistically create a complex neural and bony landscape. The torcular (opisthion of posterior fossor) is the point of greatest rearrangement in the CM II, which is typically hypoplastic, and inserts abnormally low, often placing the torcular Herophili just above the foramen magnum, especially in the edema group (Figure 1). In line with a previous study performed in postnatal patients [6], shape changes in bone and brain, as well as in the skull and ventricle, are related, further elucidating the interaction of the “core hubs” in the network. These approaches provide the algorithm basis supporting the “deformation” diathesis of CM II, indicating that the abnormal form, shape, or position of the skull, brain, venous structures, and CSF spaces is caused by mechanical forces rooted in the intrinsic malformation of spina bifida. This aligns with the view of experts who have shifted from labeling it a malformation to referring to it as a deformation sequence [7, 44, 45]. While debate exists as to whether corpus callosal dysgenesis is due to hydrocephalus or is inherently malformed [7, 11], our results show that landmarks of the corpus callosum are induced, at least in part, by a set of mechanical effects of the spine/skull deformity.

Regarding the higher incidence rate of brain edema in older fetuses (Table S2), we conducted a detailed analysis of the influence of gestational age on these mechanical events (Figure 5) [10, 46]. The outer CSF spaces exhibited a decreasing trend, while the degree of vermis ectopia exacerbated with increased GA. In addition, we noted that mildly or moderately enlarged ventriculomegaly (depicted as a larger node in Figure 3A) with pointed occipital horns and a lemon‐shaped head, along with a smaller head circumference, are more common in the early gestational stages (see Figures S1 and S2). In contrast, severe ventriculomegaly/hydrocephalus (depicted as a smaller node) and brain edema with a larger head circumference become more prevalent in later gestational weeks (Figures 1 and 2). This suggests that the progression of brain edema is possibly a time‐dependent event. The reason may be the continuous worsening due to altered CSF dynamics, which results in an accumulation of adverse structural alterations to the skull, hindbrain, and finally, the vasculature, with, ultimately, a white matter rearrangement at the developmental fetal stage. As crowding progresses in the “fixed” posterior fossa over time, with an oppositely expanding brain parenchyma, the dural venous structure is attached to the crista galli just beneath the cranial vault [47, 48], which could be compressed and gradually become stenotic, leading to deep vein congestion (Figure 1) and vasogenic edema.

Our study has several limitations. First, due to the single‐center nature of the study, identical cohorts could not be followed from pre‐ to postnatal stages using phenome analysis due to termination of pregnancy in several of our cases. However, given the reasonable amount of included pre‐ and postnatal high‐quality imaging data, no great impact on the phenome appearances is expected in larger cohorts. Second, shape comparisons between fetuses with and without edema were constrained on an age‐matched basis, resulting in a relatively small sample size. However, it remains suitable as previously acknowledged in the literature. Third, it should be noted that the data used in this study were cross‐sectional, which limits the ability to observe dynamic changes over time; future longitudinal studies might need to elucidate these time‐dependent events. Given the retrospective nature of this study, there is a need for a prospective multicenter study to validate these findings and to evaluate the impact of fetal surgery on this condition.

In conclusion, network medicine and biometric analysis together characterize in utero phenotypic unfolding of CM II and provide diverse insights into the origin and characterization of distinct phenotypes amongst this condition. Brain edema is identified as a more severe phenotype with a distinct anatomical/shape origin. These approaches support and validate the pathogenic “deformation” sequence nature of this complex syndrome, which primarily stems from a primary defect: spina bifida malformation. This further supports the rationale for prenatal surgery, which aims to repair the spinal defect and prevent ongoing deformation events.

## Ethics Statement

This retrospective single‐center study was approved by the institutional review board of the Medical University of Vienna on 27.11.2017 (Ethics Committee number 1716/2017).

## Consent

Written informed consent was waived by the Institutional Review Board.

## Conflicts of Interest

The authors of this manuscript declare no relationships with any companies whose products or services may be related to the subject matter of the article.

## Supporting information

Supporting Information S1

## Data Availability

The data supporting the findings of this study are available from the corresponding author upon reasonable request.
