# Supplementary material for: Characterizing the In Utero Phenome of the Chiari II Malformation—A Network Medicine Approach, Using Fetal MRI
Source: Prenat Diagn. 2025 Jan 3;45(3):362–73. doi: 10.1002/pd.6741 (PMC11893518; doi:10.1002/pd.6741)
Supplement: Supplementary file 1 — Supporting Information S1 [file PD-45-362-s001.docx]

**Supplementary Material**

**
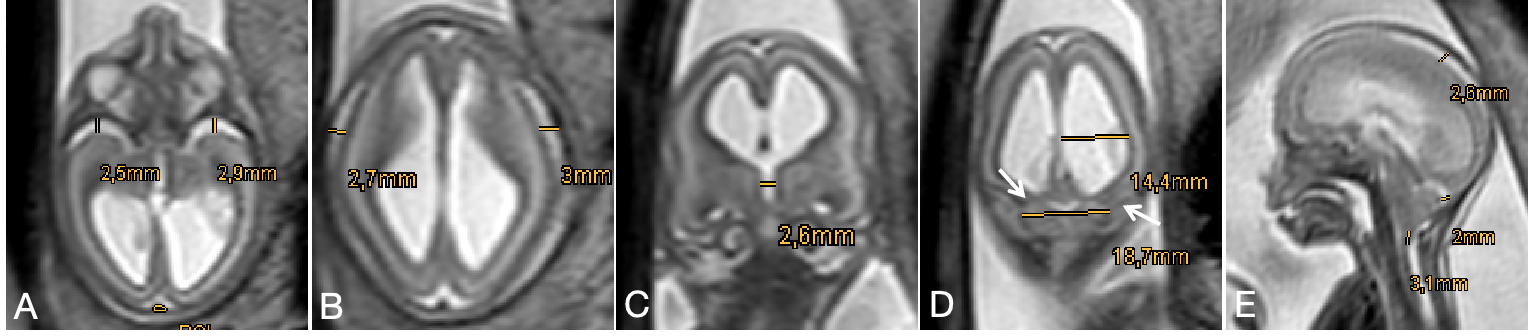
**

**Figure 1:** Example of variant measurement via PACS tools in a fetus with Chiari II Malformation at GA 23+4 weeks.

A: Superior sagittal sinus was calculated by averaging three slices’ measurements above the torcular level, delineating the flow void; T2-SSFSE sequence.

A, B, and E: Outer cerebrospinal fluid spaces were calculated by summarizing the insular cistern and temporobasal cistern (axial plane), as well as the central and retrocerebellar subarachnoid spaces (mid-sagittal plane).

C, D: The atrium width of the lateral ventricle and third ventricle width were measured in the coronal plane.

D: To measure the maximum diameter of the posterior fossa, the distance between the medial surfaces of the lateral bony margins of the posterior fossa at the level of the lateral insertions of the tentorium cerebelli (arrows) was depicted on coronal T2-weighted SSFSE sequences.

E: The caudal extent of the vermis ectopia was measured beginning from the foramen magnum.

**
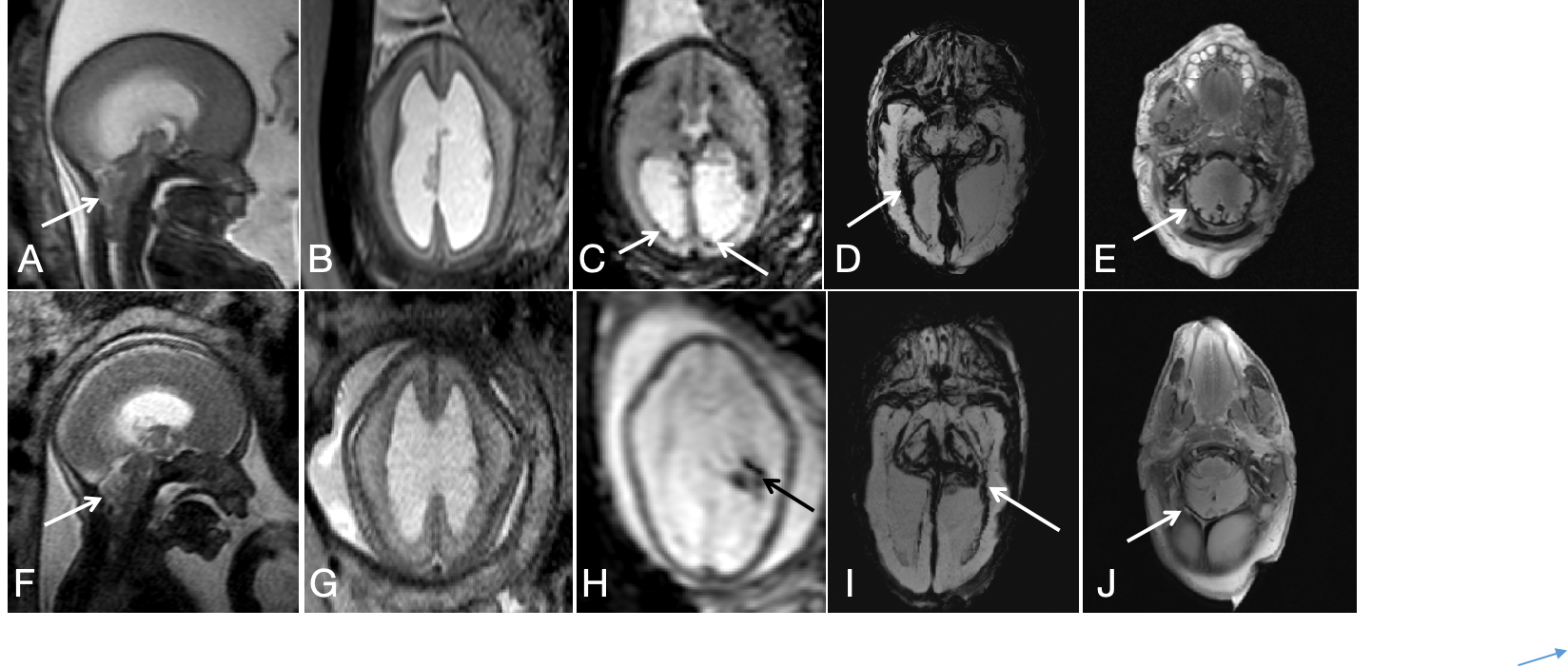
Figure 2:** Fetuses with Chiari II malformation and ICH demonstrated congested deep veins on postmortem image.

A-E: GA22+1weeks, A: There is a significant downward displacement of the vermis cerebelli, extending to the level of C5 (white arrow). The outer cerebrospinal fluid spaces within the posterior cranial fossa are fully occupied. B: The planimetric measurement of the skull circumference on MRI was 187 mm, corresponding to the 30th percentile. Outer CSF spaces were depleted with brain parenchyma edema (high signal intensity). C: Blood-sensitive sequences reveal congested subependymal veins with small ependymal hemorrhage (white arrows). D-E: A postmortem MRI was conducted seven days later, revealing pronounced congestion in the plexus and subependymal deep veins, as evident on susceptibility-weighted imaging (SWI). Congested inferior vermis vein and the narrowing transverse sinus (white arrow) behind it as shown on T2-CISS sequence.

F-J: GA22+3, F-G: A pronounced Chiari II malformation is evident, accompanied by the characteristic lemon-head shape. The posterior cranial fossa is notably undersized, and a deep tentorium is observed. Portions of the cerebellum extend into the spinal canal, reaching approximately up to the level of C4 (arrow). H: Hemorrhage was observed on the left side of the ependyma and adjacent plexus. I-J: Subsequent postmortem images reveal stenosis of the transverse sinus and congestion within the deep veins and subependymal veins along the left occipital horn.


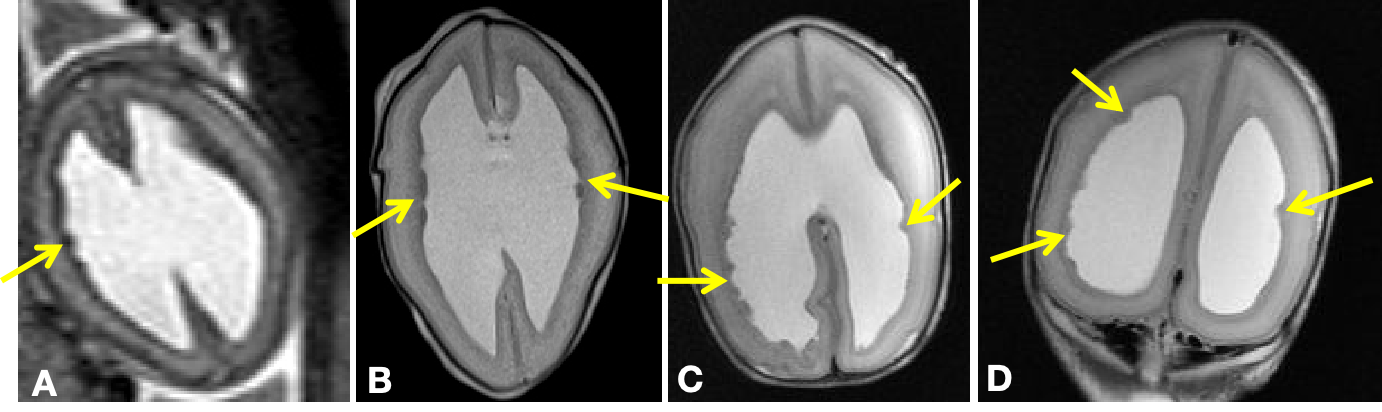


**Figure 3:** Prenatally detected heterotopia in a CM II fetus, confirmed by postmortem MRI.

A: T2 FSE image shows subependymal heterotopia nodules in a CM II fetus at GA 20+0. B-D: Postmortem image of the same fetus performed at 23+0 weeks showed more subependymal heterotopia nodules (yellow arrows).


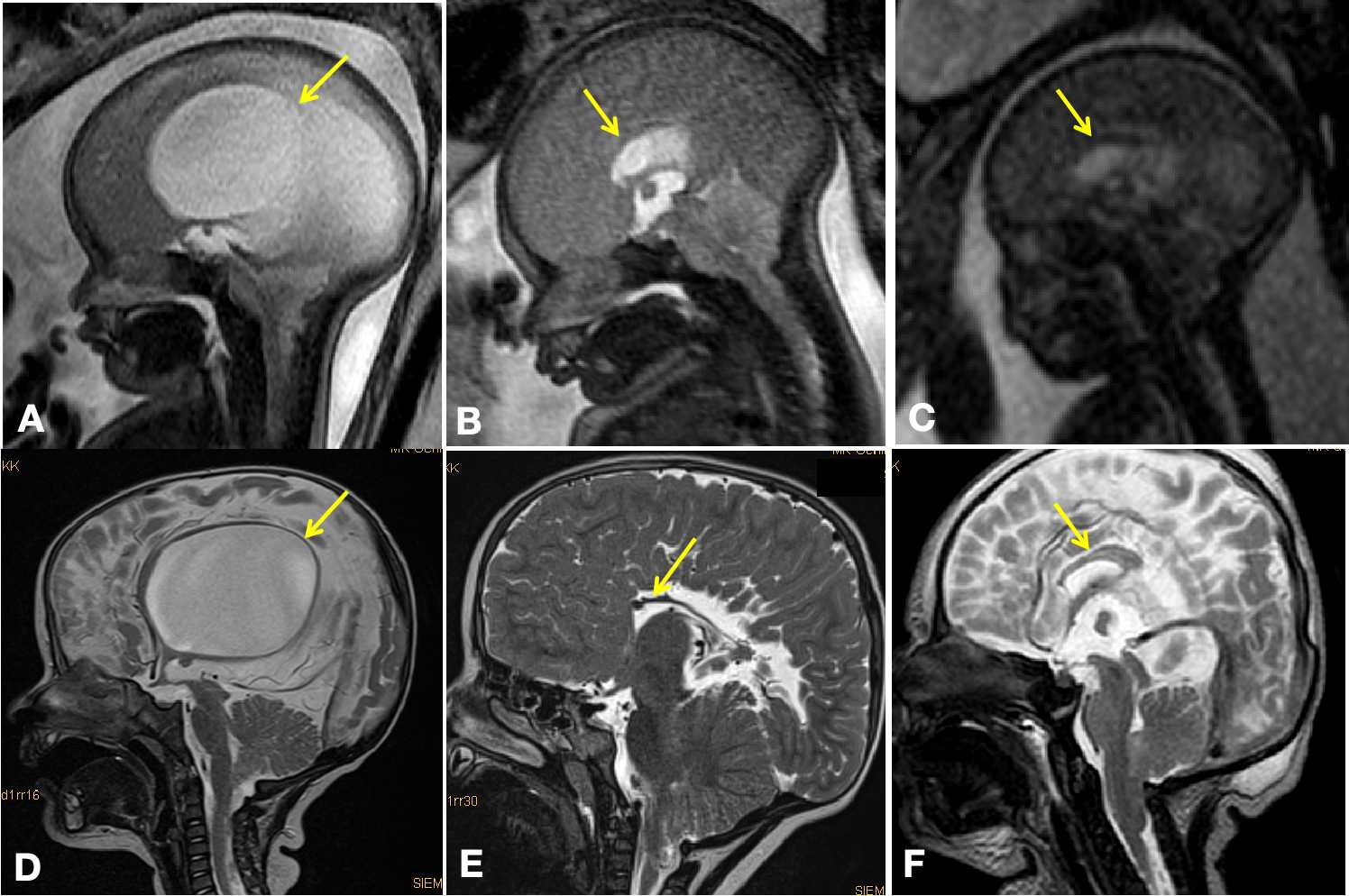


Figure 4: Follow-up postnatal MRI of fetuses with Chiari II malformation confirmed prenatally suspected callosal dysgenesis.

(A, B, C) Prenatal MRI showed suspected callosal dysgenesis at GW28+1, 34+0, and 29+5, respectively. (D, E, F) Postnatal MRI was conducted seven weeks, 20, and 18 months after birth, respectively, demonstrating callosal dysgenesis^1^—thin, irregular shape and lack of entirety (arrow).


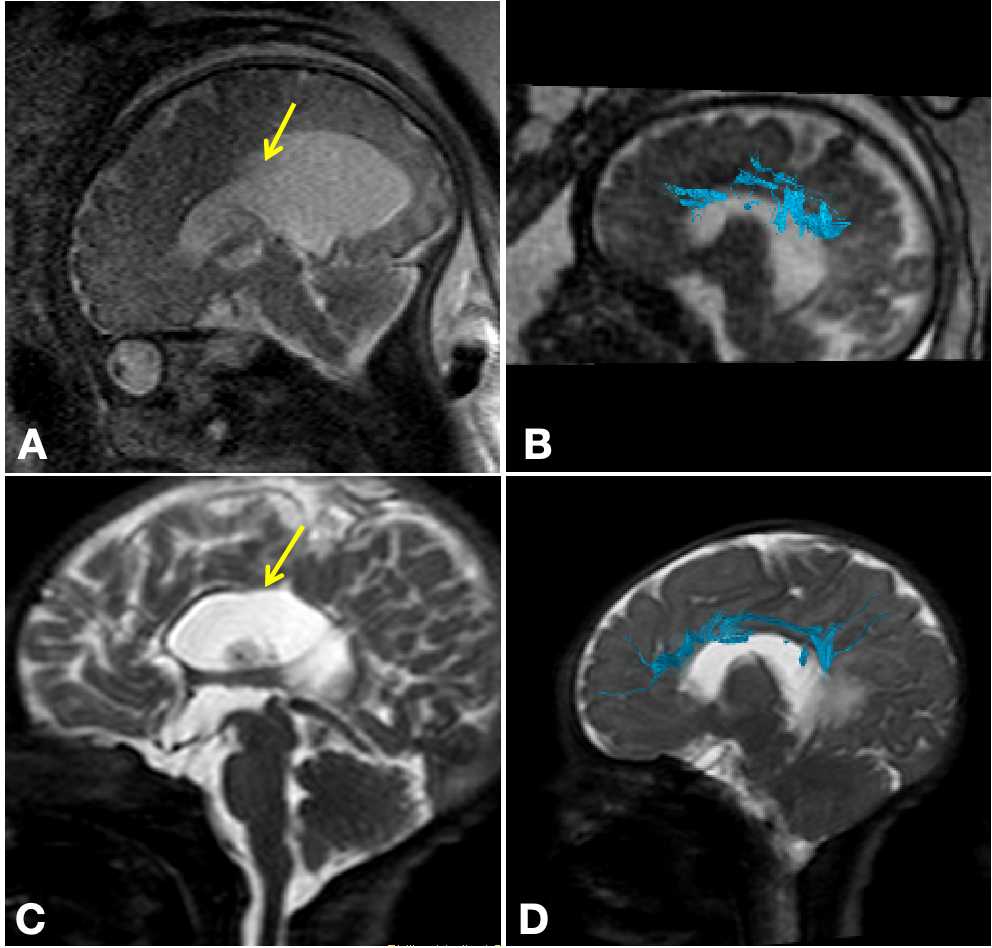


**Figure 5:** Examples of Chiari II cases demonstrate callosal dysgenesis, as supported by both pre-and postnatal DTI findings.

(A, B) Prenatal MRI showed suspected callosal dysgenesis at GW34+0. (C, D) Postnatal MRI was conducted 14 months after birth, demonstrating the abnormal callosal—thin, irregular shape and lack of entirety (arrow). (B, D) DTI images show disrupted and disorganized fibers at the corpus callosal.


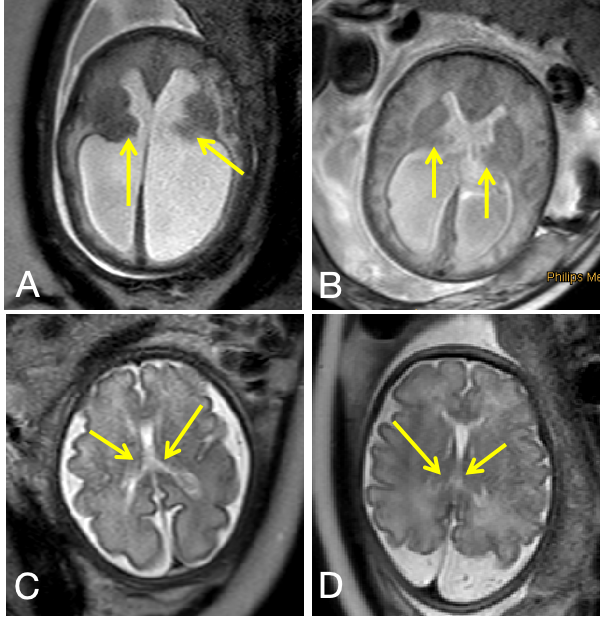


**Figure 6:** Examples of Chiari II cases present large massa intermedia(arrows) at GA 33+3 weeks(A) and 34+5 weeks(B) and their age-matched normal cases (C, GA 33+1 weeks; D, GA 34+5 weeks) as a comparison.


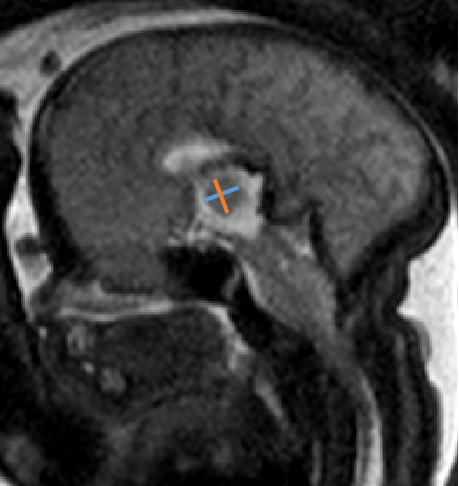


**Figure 7:** Example of massa intermedia measurement in a CM II fetus at GA 33+3 weeks.

The blue line represents the anteroposterior diameter, the orange line represents the craniocaudal diameter, and the area of the massa intermedia is multiplied by the above and recorded for comparison.


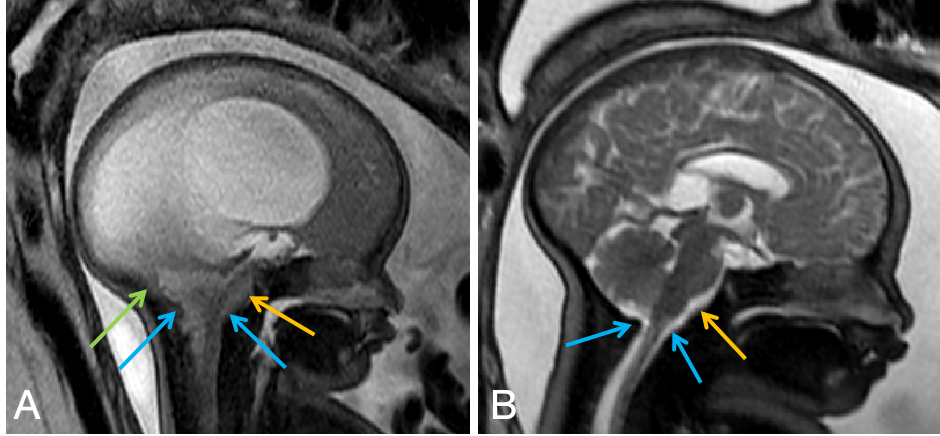


**Figure 8:** Examples of Chiari II cases (A, B) present short clivus (orange arrows) accompanied by a low-lying torcular herophili (green arrow) and enlarged foramen magnum (blue arrows) at GA 34+3 weeks compared to the age-matched normal case at GA 34+5.


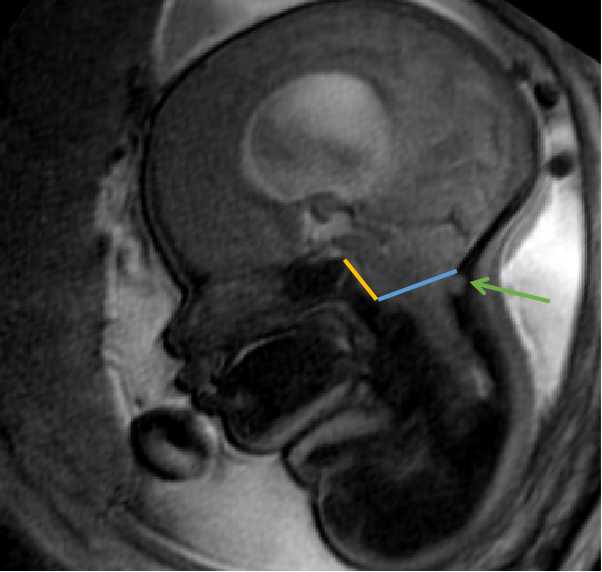


**Figure 9:** Example of foramen magnum clivus length measurement in a CM II fetus at GA 35 weeks.

The orange line represents the clivus length measurement, and the blue line is the McRae line^2^, or basion-opisthion line, which extends from the anterior margin of the foramen magnum (basion) to its posterior margin (opisthion). The protrusion of the atlantooccipital membrane (green arrow), which lies just below the foramen magnum, could further aid as a reference to measure the foramen magnum.

.
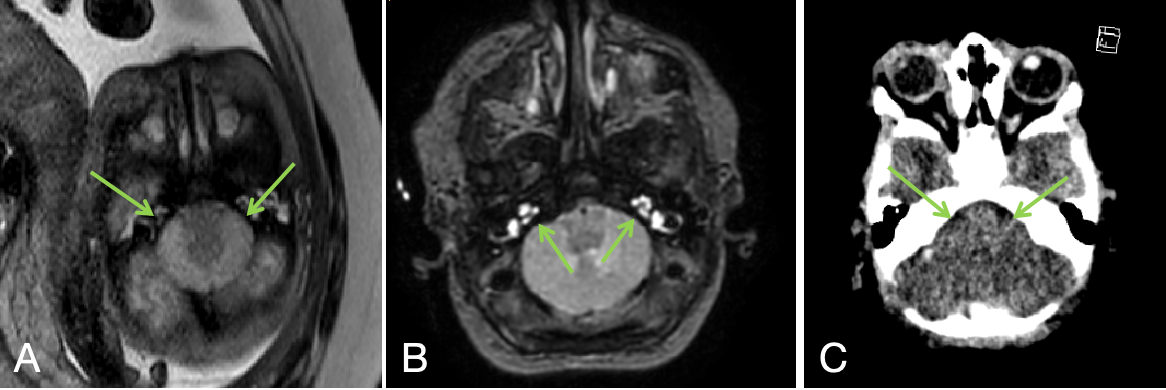


**Figure 10:** Example of a Chiari II case demonstrating scalloping of the petrous bone (green arrows) on prenatal MRI (A) and follow-up postnatal MRI(B) and CT(C).

**
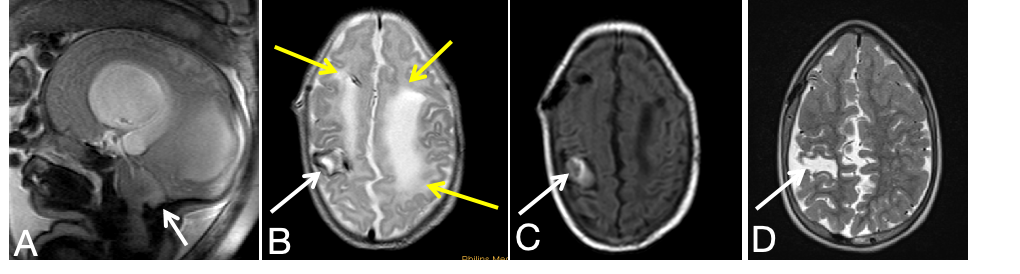
**

**Figure 11:** Follow-up postnatal MRI of fetuses with Chiari II malformation and brain edema show intracranial hemorrhage and brain tissue loss/defect.

(A) Prenatal MRI showed pronounced edema at GW34+0. (B, C) Postnatal MRI was conducted seven weeks after birth, revealing the presence of intracranial hemorrhages in the right post-central parenchyma (white arrow), and residual edema (yellow arrows). (D) Postnatal MRI performed 1 year after birth shows brain tissue loss/defect at the site of previous hemorrhage(arrow).


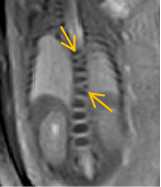


**Figure 12:** T2 SSFP image shows multiple wedged hemivertebra (orange arrow) in a CM II fetus at GA 30 weeks.


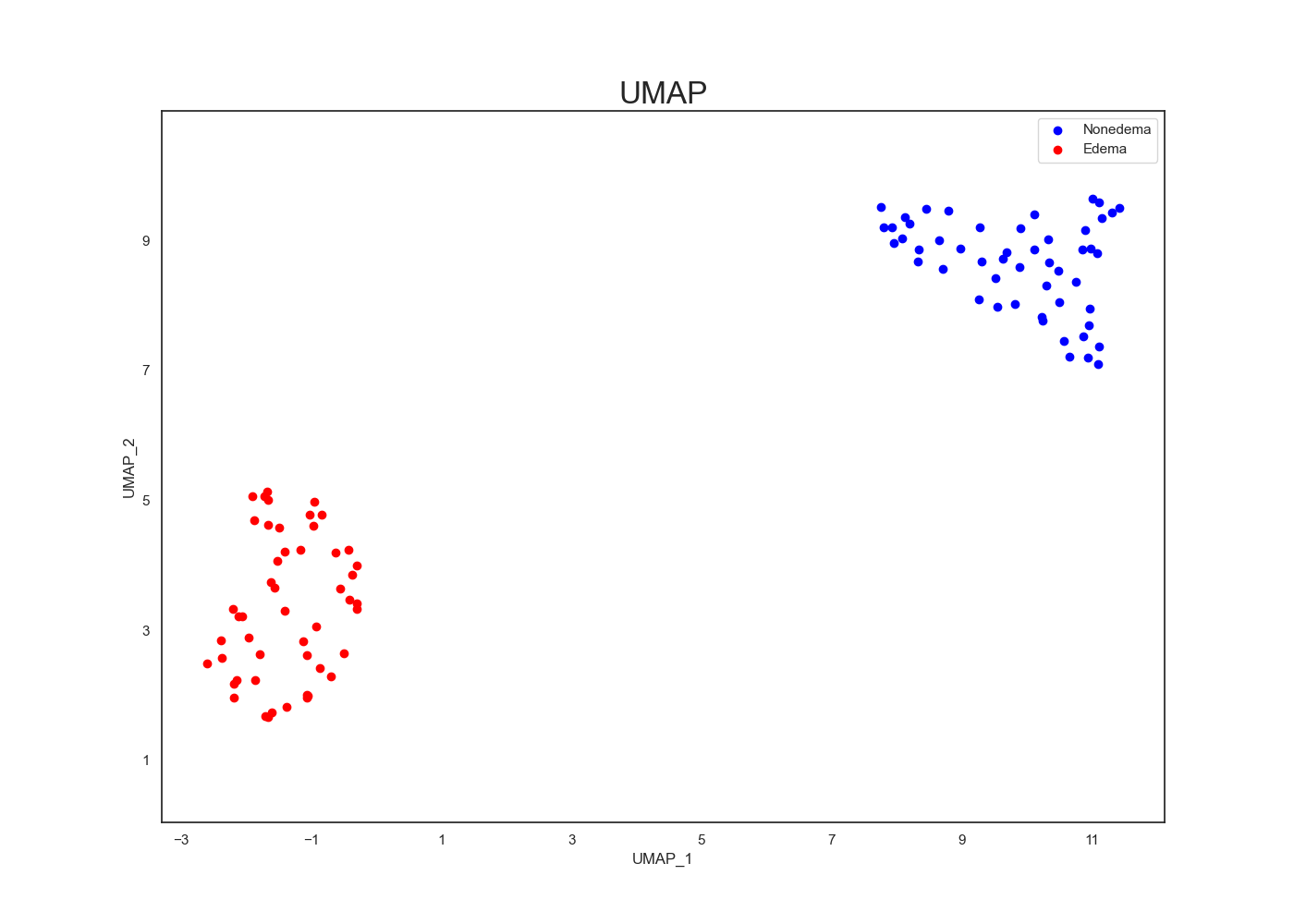


**Figure 13:** 2D UMAP dimensional reduction analysis illustrates distinct cluster patterns between the CM II with and without edema subtypes.


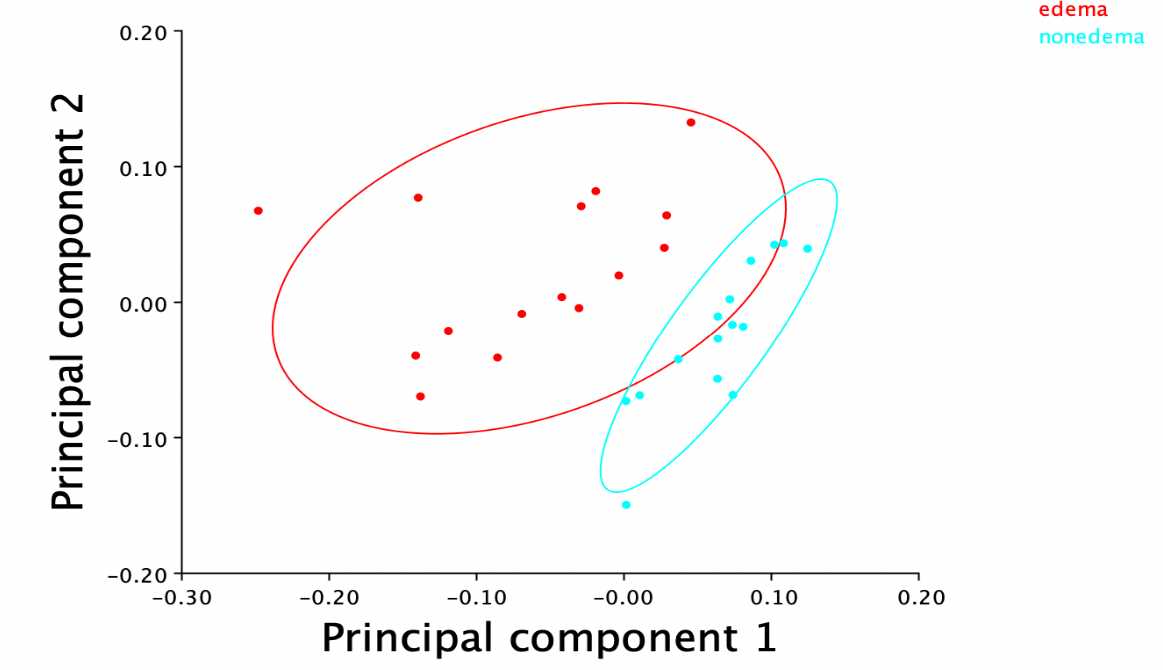


**Figure 14:** Principal component (relative wrap) plot of CM II cases with and without brain edema. Principal components are optimally powerful explanatory factors of shape variability. The approximate position of the centroid of the edema group is marked in red and the nonedema group is marked in blue.

| Supplementary Table 1 Qualitative and quantitative MR assessment in Chiari II Malformations | | | | |  |
| --- | --- | --- | --- | --- | --- |
| Abnormality | **No. of fetuses(*n*=91)**  **No. of Presence Interrater agreement**  **κ values** | | **Quantitative measurements**  **CM II Normal controls^a^ P-value**  **Mean SD Mean SD** | | |
| Supratentorial brain structure: |  |  |  |  |  |
| Brain edema | 50 | 0.88 |  |  |  |
| Falx defect | 65 | 0.96 |  |  |  |
| Callosal dysgenesis | 60 | 0.93 |  |  |  |
| Subependymal heterotopia | 24 | 0.89 |  |  |  |
| Large massa intermedia^c^ (mm^2^) |  |  | 35.3 6.4 | 21.5 5.6 | 0.001 |
| Cortical development delay | 8 | 0.93 |  |  |  |
| Infratentorial brain structure: |  |  |  |  |  |
| medulla oblongata kinking | 57 | 0.96 |  |  |  |
| vermis ectopia | 91 | 0.97 |  |  |  |
| prutrusion of alantooccipital membrane | 64 | 0.97 |  |  |  |
| low-lying tentorium | 19 | 0.97 |  |  |  |
| beaking of the tectum | 61 | 0.96 |  |  |  |
| Skull: |  |  |  |  |  |
| lemon head | 53 | 0.97 |  |  |  |
| scalloping of the petrous bone | 29 | 0.90 |  |  |  |
| small posterior fossa_(mm)_^c^ |  |  | 22.5 4.4 | 30.1 4.5 | <0.001 |
| foramen magnum enlargement_(mm)_^c^ |  |  | 17.4 5.6 | 14.3 5.9 | <0.001 |
| shortening of the clivus_(mm)_^c^ |  |  | 13.7 3.7 | 16.4 3.5 | 0.005 |
| CSF system: |  |  |  |  |  |
| Ventriculomegaly by grading^b^: |  |  |  |  |  |
| Mild/moderate VM | 51 | 0.97 |  |  |  |
| Severe VM/hydrocephalus | 40 | 0.98 |  |  |  |
| aqueduct stenosis | 54 | 0.93 |  |  |  |
| 4th ventricle stenosis | 46 | 0.95 |  |  |  |
| third ventricle widening | 37 | 0.95 |  |  |  |
| reduced outer CSF space | 67 | 0.96 |  |  |  |
| pointed occipital horn | 34 | 0.94 |  |  |  |
| Vascular structure: |  |  |  |  |  |
| intracranial hemorrhage | 44 | 0.95 |  |  |  |
| venous sinus stenosis | 43 | 0.88 |  |  |  |
| venous congestion | 42 | 0.88 |  |  |  |
| Spine: |  |  |  |  |  |
| spinal bony defect | 91 | 0.98 |  |  |  |
| scoliosis | 17 | 0.95 |  |  |  |
| hydromyelia | 15 | 0.89 |  |  |  |
| wedge/hemivertebrae | 19 | 0.89 |  |  |  |
| syringomyelia | 17 | 0.93 |  |  |  |
| diastematomyelia | 13 | 0.91 |  |  |  |
| ^a^ average by 2 normal age-matched fetuses; ^b^ maximum atrium width, grading as mild (10-12mm), moderate (12-15mm), severe(>15mm); ^c^ see Supplementary Material for measurement details | | | | | |

| Supplementary Table 2 Fetal demographics and MRI characteristics | |
| --- | --- |
| Characteristics | **All fetuses(*n*=91)** |
| Fetal characteristics |  |
| Number of fetuses | **91** |
| MRI |  |
| Number of MRI scans | **101** |
| Median gestational age at fetal MRI | **24.4(16.7-37.3)** |
| Number of fetal MRI scans |  |
| 1 | **83** |
| 2 | **6** |
| 3 | **2** |
| Incidence of brain edema (%) |  |
| All gestational age | **43/91 (47.2)** |
| ≤26 weeks (%) | **20/56(35.7)** |
| >26 weeks (%) | **23/35(65.7)** |

| Supplementary Table 3 The top 10 ranking of the network features | | | |
| --- | --- | --- | --- |
| Ranking | **Closeness centrality** | **Betweenness centrality** | **Degree centrality** |
| 1 | Spinal bony defect | Spinal bony defect | Spinal bony defect |
| 2 | Small posterior fossa | Small posterior fossa | Small posterior fossa |
| 3 | Vermis ectopia | Vermis ectopia | Vermis ectopia |
| 4 | Reduced CSF spaces | Reduced CSF spaces | Reduced CSF spaces |
| 5 | Adequate stenosis | Adequate stenosis | Adequate stenosis |
| 6 | Hydrocephalus | Protrusion of atlantooccipital  membrane | Hydrocephalus |
| 7 | Protrusion of atlantooccipital membrane | Hydrocephalus | Protrusion of atlantooccipital membrane |
| 8 | Venous sinus stenosis | Venous sinus stenosis | Falx defect |
| 9 | Venous congestion | Venous congestion | Venous congestion |
| 10 | Falx defect | Falx defect | Venous sinus stenosis |

1. Khalaveh F, Seidl R, Czech T, et al. Myelomeningocele-Chiari II malformation-Neurological predictability based on fetal and postnatal magnetic resonance imaging. *Prenat Diagn.* 2021;41(8):922-932.

2. Hofmann E, Prescher A. The clivus: anatomy, normal variants and imaging pathology. *Clin Neuroradiol.* 2012;22(2):123-139.
